# Supplementary material for: Modeling glioblastoma heterogeneity as a dynamic network of cell states
Source: Mol Syst Biol. 2021 Sep 16;17(9):e10105. doi: 10.15252/msb.202010105 (PMC8444284; doi:10.15252/msb.202010105)
Supplement: Supplementary file 6 — Source Data for Figure 5 [file MSB-17-e10105-s004.zip › Figure5A_sourcedata/GSEA_3071/nosigdb_stateV.GseaPreranked.1622795159954/FELDMAN_2020_G2-M.html]

Details for gene set FELDMAN\_2020\_G2-M[GSEA]

|  || Dataset | state43071 |
| Phenotype | NoPhenotypeAvailable |
| Upregulated in class | na\_pos |
| GeneSet | FELDMAN\_2020\_G2-M |
| Enrichment Score (ES) | 0.5704608 |
| Normalized Enrichment Score (NES) | 3.5653937 |
| Nominal p-value | 0.0 |
| FDR q-value | 0.0 |
| FWER p-Value | 0.0 |
Table: GSEA Results Summary

  

Fig 1: Enrichment plot: FELDMAN\_2020\_G2-M      
 Profile of the Running ES Score & Positions of GeneSet Members on the Rank Ordered List

  

| PROBE | GENE SYMBOL | GENE\_TITLE | RANK IN GENE LIST | RANK METRIC SCORE | RUNNING ES | CORE ENRICHMENT || 1 | ESCO2 |  |  | 3 | 2.193 | 0.0100 | Yes |
| 2 | HMGB2 |  |  | 5 | 2.011 | 0.0194 | Yes |
| 3 | NUSAP1 |  |  | 6 | 2.001 | 0.0291 | Yes |
| 4 | PTPRZ1 |  |  | 13 | 1.865 | 0.0369 | Yes |
| 5 | TOP2A |  |  | 14 | 1.842 | 0.0457 | Yes |
| 6 | NCAPG |  |  | 21 | 1.760 | 0.0530 | Yes |
| 7 | H2AFX |  |  | 26 | 1.725 | 0.0605 | Yes |
| 8 | CENPF |  |  | 28 | 1.705 | 0.0685 | Yes |
| 9 | RRM2 |  |  | 34 | 1.660 | 0.0755 | Yes |
| 10 | MKI67 |  |  | 45 | 1.598 | 0.0812 | Yes |
| 11 | FOXM1 |  |  | 53 | 1.550 | 0.0873 | Yes |
| 12 | PBK |  |  | 54 | 1.536 | 0.0947 | Yes |
| 13 | MXD3 |  |  | 62 | 1.500 | 0.1005 | Yes |
| 14 | GTSE1 |  |  | 66 | 1.491 | 0.1071 | Yes |
| 15 | CDK1 |  |  | 69 | 1.488 | 0.1139 | Yes |
| 16 | PTN |  |  | 70 | 1.482 | 0.1210 | Yes |
| 17 | SMC2 |  |  | 73 | 1.466 | 0.1277 | Yes |
| 18 | SPC25 |  |  | 76 | 1.442 | 0.1342 | Yes |
| 19 | IGFBP3 |  |  | 78 | 1.437 | 0.1409 | Yes |
| 20 | UBE2T |  |  | 79 | 1.432 | 0.1478 | Yes |
| 21 | FBXO5 |  |  | 82 | 1.422 | 0.1543 | Yes |
| 22 | SCRG1 |  |  | 87 | 1.404 | 0.1602 | Yes |
| 23 | HES6 |  |  | 91 | 1.391 | 0.1663 | Yes |
| 24 | DEK |  |  | 94 | 1.384 | 0.1726 | Yes |
| 25 | CDCA2 |  |  | 96 | 1.382 | 0.1790 | Yes |
| 26 | FABP7 |  |  | 98 | 1.372 | 0.1854 | Yes |
| 27 | KIFC1 |  |  | 113 | 1.338 | 0.1891 | Yes |
| 28 | CENPM |  |  | 114 | 1.337 | 0.1955 | Yes |
| 29 | TACC3 |  |  | 116 | 1.334 | 0.2018 | Yes |
| 30 | GPM6B |  |  | 121 | 1.326 | 0.2073 | Yes |
| 31 | SMC4 |  |  | 127 | 1.320 | 0.2127 | Yes |
| 32 | RTKN2 |  |  | 143 | 1.285 | 0.2159 | Yes |
| 33 | GMNN |  |  | 146 | 1.280 | 0.2217 | Yes |
| 34 | NCAPD2 |  |  | 157 | 1.252 | 0.2257 | Yes |
| 35 | MIS18BP1 |  |  | 159 | 1.247 | 0.2315 | Yes |
| 36 | TMPO |  |  | 161 | 1.243 | 0.2373 | Yes |
| 37 | CENPK |  |  | 162 | 1.241 | 0.2433 | Yes |
| 38 | CDKN3 |  |  | 163 | 1.239 | 0.2492 | Yes |
| 39 | LMNB1 |  |  | 165 | 1.238 | 0.2550 | Yes |
| 40 | KIF23 |  |  | 167 | 1.235 | 0.2607 | Yes |
| 41 | CDCA3 |  |  | 169 | 1.230 | 0.2665 | Yes |
| 42 | GGH |  |  | 182 | 1.207 | 0.2699 | Yes |
| 43 | DBF4 |  |  | 189 | 1.197 | 0.2745 | Yes |
| 44 | KIF4A |  |  | 191 | 1.193 | 0.2800 | Yes |
| 45 | NUF2 |  |  | 192 | 1.189 | 0.2857 | Yes |
| 46 | ZWINT |  |  | 204 | 1.166 | 0.2892 | Yes |
| 47 | ASPM |  |  | 207 | 1.163 | 0.2944 | Yes |
| 48 | PRC1 |  |  | 211 | 1.161 | 0.2994 | Yes |
| 49 | ARL6IP1 |  |  | 212 | 1.158 | 0.3049 | Yes |
| 50 | KIF22 |  |  | 213 | 1.157 | 0.3105 | Yes |
| 51 | UBE2C |  |  | 214 | 1.154 | 0.3161 | Yes |
| 52 | CDKN2C |  |  | 223 | 1.143 | 0.3200 | Yes |
| 53 | NDC80 |  |  | 240 | 1.129 | 0.3222 | Yes |
| 54 | TNC |  |  | 246 | 1.123 | 0.3267 | Yes |
| 55 | TPX2 |  |  | 248 | 1.122 | 0.3319 | Yes |
| 56 | RAD21 |  |  | 256 | 1.109 | 0.3358 | Yes |
| 57 | LIMA1 |  |  | 264 | 1.098 | 0.3397 | Yes |
| 58 | FGFBP3 |  |  | 268 | 1.093 | 0.3444 | Yes |
| 59 | KIF14 |  |  | 272 | 1.090 | 0.3490 | Yes |
| 60 | BUB1 |  |  | 277 | 1.084 | 0.3534 | Yes |
| 61 | CKAP2L |  |  | 285 | 1.078 | 0.3572 | Yes |
| 62 | BEX1 |  |  | 287 | 1.077 | 0.3622 | Yes |
| 63 | ECT2 |  |  | 295 | 1.071 | 0.3660 | Yes |
| 64 | AURKB |  |  | 297 | 1.068 | 0.3709 | Yes |
| 65 | ARHGAP11A |  |  | 303 | 1.064 | 0.3751 | Yes |
| 66 | STMN1 |  |  | 307 | 1.062 | 0.3796 | Yes |
| 67 | CCNA2 |  |  | 309 | 1.057 | 0.3845 | Yes |
| 68 | CKAP2 |  |  | 312 | 1.053 | 0.3892 | Yes |
| 69 | PRR11 |  |  | 317 | 1.048 | 0.3934 | Yes |
| 70 | RHNO1 |  |  | 326 | 1.040 | 0.3968 | Yes |
| 71 | TMEM106C |  |  | 334 | 1.034 | 0.4004 | Yes |
| 72 | S100B |  |  | 361 | 0.996 | 0.4001 | Yes |
| 73 | CDKN1A |  |  | 363 | 0.993 | 0.4046 | Yes |
| 74 | KIF11 |  |  | 369 | 0.982 | 0.4084 | Yes |
| 75 | PHF19 |  |  | 378 | 0.971 | 0.4115 | Yes |
| 76 | TTK |  |  | 383 | 0.966 | 0.4153 | Yes |
| 77 | DEPDC1B |  |  | 387 | 0.963 | 0.4194 | Yes |
| 78 | NOVA1 |  |  | 393 | 0.957 | 0.4230 | Yes |
| 79 | RPA3 |  |  | 395 | 0.953 | 0.4274 | Yes |
| 80 | HJURP |  |  | 406 | 0.945 | 0.4299 | Yes |
| 81 | SPC24 |  |  | 414 | 0.938 | 0.4331 | Yes |
| 82 | NUDT1 |  |  | 417 | 0.936 | 0.4372 | Yes |
| 83 | DLGAP5 |  |  | 418 | 0.936 | 0.4417 | Yes |
| 84 | MAD2L1 |  |  | 421 | 0.932 | 0.4458 | Yes |
| 85 | KPNA2 |  |  | 435 | 0.919 | 0.4476 | Yes |
| 86 | CENPE |  |  | 437 | 0.918 | 0.4518 | Yes |
| 87 | LBR |  |  | 438 | 0.916 | 0.4562 | Yes |
| 88 | RACGAP1 |  |  | 443 | 0.912 | 0.4598 | Yes |
| 89 | FAM83D |  |  | 447 | 0.909 | 0.4636 | Yes |
| 90 | CENPH |  |  | 448 | 0.908 | 0.4680 | Yes |
| 91 | METRN |  |  | 450 | 0.907 | 0.4722 | Yes |
| 92 | KNSTRN |  |  | 457 | 0.902 | 0.4753 | Yes |
| 93 | CLU |  |  | 464 | 0.900 | 0.4785 | Yes |
| 94 | SHCBP1 |  |  | 465 | 0.900 | 0.4828 | Yes |
| 95 | HMGN2 |  |  | 466 | 0.900 | 0.4871 | Yes |
| 96 | POC1A |  |  | 467 | 0.896 | 0.4914 | Yes |
| 97 | LSM3 |  |  | 468 | 0.892 | 0.4957 | Yes |
| 98 | PCSK1N |  |  | 476 | 0.887 | 0.4986 | Yes |
| 99 | SPAG5 |  |  | 481 | 0.884 | 0.5021 | Yes |
| 100 | MPHOSPH9 |  |  | 489 | 0.877 | 0.5049 | Yes |
| 101 | UBE2S |  |  | 514 | 0.854 | 0.5043 | Yes |
| 102 | PLK1 |  |  | 526 | 0.850 | 0.5062 | Yes |
| 103 | BUB1B |  |  | 535 | 0.846 | 0.5087 | Yes |
| 104 | BIRC5 |  |  | 541 | 0.842 | 0.5117 | Yes |
| 105 | CDC25B |  |  | 566 | 0.820 | 0.5109 | Yes |
| 106 | CMC2 |  |  | 580 | 0.812 | 0.5123 | Yes |
| 107 | HMGB1 |  |  | 583 | 0.810 | 0.5158 | Yes |
| 108 | DIAPH3 |  |  | 585 | 0.808 | 0.5195 | Yes |
| 109 | TK1 |  |  | 591 | 0.806 | 0.5223 | Yes |
| 110 | TTYH1 |  |  | 611 | 0.794 | 0.5224 | Yes |
| 111 | OIP5 |  |  | 620 | 0.788 | 0.5246 | Yes |
| 112 | HMMR |  |  | 622 | 0.788 | 0.5282 | Yes |
| 113 | CENPA |  |  | 627 | 0.786 | 0.5312 | Yes |
| 114 | PIF1 |  |  | 632 | 0.782 | 0.5342 | Yes |
| 115 | SKA3 |  |  | 668 | 0.765 | 0.5309 | Yes |
| 116 | KIF18A |  |  | 679 | 0.759 | 0.5326 | Yes |
| 117 | GAS2L3 |  |  | 685 | 0.755 | 0.5352 | Yes |
| 118 | ANP32E |  |  | 722 | 0.739 | 0.5317 | Yes |
| 119 | MAD2L2 |  |  | 724 | 0.736 | 0.5350 | Yes |
| 120 | CKAP5 |  |  | 752 | 0.723 | 0.5332 | Yes |
| 121 | AURKA |  |  | 761 | 0.718 | 0.5350 | Yes |
| 122 | DCP2 |  |  | 762 | 0.717 | 0.5385 | Yes |
| 123 | TUBG1 |  |  | 776 | 0.712 | 0.5393 | Yes |
| 124 | PSRC1 |  |  | 787 | 0.705 | 0.5407 | Yes |
| 125 | SNRNP25 |  |  | 795 | 0.702 | 0.5427 | Yes |
| 126 | DTYMK |  |  | 800 | 0.700 | 0.5453 | Yes |
| 127 | TRIP13 |  |  | 804 | 0.698 | 0.5481 | Yes |
| 128 | TUBB |  |  | 836 | 0.685 | 0.5452 | Yes |
| 129 | H2AFZ |  |  | 851 | 0.677 | 0.5457 | Yes |
| 130 | RNASEH2A |  |  | 859 | 0.674 | 0.5476 | Yes |
| 131 | NEK2 |  |  | 901 | 0.659 | 0.5427 | Yes |
| 132 | MELK |  |  | 915 | 0.653 | 0.5432 | Yes |
| 133 | MZT1 |  |  | 917 | 0.652 | 0.5462 | Yes |
| 134 | H2AFV |  |  | 933 | 0.647 | 0.5463 | Yes |
| 135 | KIF20B |  |  | 934 | 0.647 | 0.5494 | Yes |
| 136 | PTTG1 |  |  | 936 | 0.647 | 0.5523 | Yes |
| 137 | TMEM237 |  |  | 952 | 0.639 | 0.5524 | Yes |
| 138 | CENPN |  |  | 953 | 0.638 | 0.5555 | Yes |
| 139 | HNRNPA3 |  |  | 954 | 0.638 | 0.5586 | Yes |
| 140 | CKS2 |  |  | 966 | 0.634 | 0.5595 | Yes |
| 141 | PRCP |  |  | 983 | 0.626 | 0.5593 | Yes |
| 142 | RBP1 |  |  | 984 | 0.624 | 0.5623 | Yes |
| 143 | LRR1 |  |  | 991 | 0.623 | 0.5641 | Yes |
| 144 | RANBP1 |  |  | 999 | 0.620 | 0.5657 | Yes |
| 145 | LSM4 |  |  | 1003 | 0.619 | 0.5681 | Yes |
| 146 | TUBA1B |  |  | 1012 | 0.617 | 0.5695 | Yes |
| 147 | MIS18A |  |  | 1023 | 0.614 | 0.5705 | Yes |
| 148 | CDCA8 |  |  | 1043 | 0.610 | 0.5696 | No |
| 149 | MAP3K7CL |  |  | 1055 | 0.606 | 0.5704 | No |
| 150 | TMEM178A |  |  | 1073 | 0.601 | 0.5699 | No |
| 151 | RBMX |  |  | 1095 | 0.594 | 0.5686 | No |
| 152 | KIF20A |  |  | 1133 | 0.583 | 0.5641 | No |
| 153 | HMGB3 |  |  | 1154 | 0.577 | 0.5629 | No |
| 154 | NUCKS1 |  |  | 1178 | 0.570 | 0.5611 | No |
| 155 | DEPDC1 |  |  | 1179 | 0.569 | 0.5638 | No |
| 156 | ANP32B |  |  | 1187 | 0.566 | 0.5652 | No |
| 157 | HP1BP3 |  |  | 1198 | 0.562 | 0.5659 | No |
| 158 | PARPBP |  |  | 1210 | 0.560 | 0.5664 | No |
| 159 | EXOSC8 |  |  | 1230 | 0.553 | 0.5653 | No |
| 160 | ITM2B |  |  | 1244 | 0.547 | 0.5654 | No |
| 161 | TM7SF2 |  |  | 1273 | 0.539 | 0.5624 | No |
| 162 | CCDC167 |  |  | 1303 | 0.531 | 0.5592 | No |
| 163 | CCDC18 |  |  | 1305 | 0.530 | 0.5616 | No |
| 164 | KIF2C |  |  | 1309 | 0.530 | 0.5636 | No |
| 165 | CCNB2 |  |  | 1373 | 0.513 | 0.5536 | No |
| 166 | PDLIM4 |  |  | 1382 | 0.511 | 0.5544 | No |
| 167 | SPDL1 |  |  | 1397 | 0.507 | 0.5541 | No |
| 168 | CKS1B |  |  | 1502 | 0.479 | 0.5358 | No |
| 169 | CEP55 |  |  | 1508 | 0.479 | 0.5372 | No |
| 170 | EMC9 |  |  | 1522 | 0.477 | 0.5369 | No |
| 171 | G2E3 |  |  | 1602 | 0.459 | 0.5235 | No |
| 172 | TUBB4B |  |  | 1659 | 0.444 | 0.5145 | No |
| 173 | CALM3 |  |  | 1710 | 0.435 | 0.5067 | No |
| 174 | PSAP |  |  | 1855 | 0.410 | 0.4802 | No |
| 175 | CENPW |  |  | 1959 | 0.395 | 0.4617 | No |
| 176 | MEST |  |  | 1980 | 0.391 | 0.4596 | No |
| 177 | LSM5 |  |  | 1982 | 0.391 | 0.4613 | No |
| 178 | MALAT1 |  |  | 1988 | 0.391 | 0.4622 | No |
| 179 | ANLN |  |  | 2125 | 0.371 | 0.4371 | No |
| 180 | NUP35 |  |  | 2127 | 0.370 | 0.4387 | No |
| 181 | RHEB |  |  | 2192 | 0.363 | 0.4277 | No |
| 182 | HNRNPDL |  |  | 2203 | 0.362 | 0.4275 | No |
| 183 | NUDCD2 |  |  | 2311 | 0.348 | 0.4080 | No |
| 184 | CCNB1 |  |  | 2480 | 0.325 | 0.3763 | No |
| 185 | HNRNPA2B1 |  |  | 2523 | 0.320 | 0.3696 | No |
| 186 | DCXR |  |  | 2647 | 0.305 | 0.3467 | No |
| 187 | RNF26 |  |  | 2660 | 0.303 | 0.3458 | No |
| 188 | SRSF3 |  |  | 2732 | 0.297 | 0.3331 | No |
| 189 | HMGN1 |  |  | 2742 | 0.295 | 0.3328 | No |
| 190 | SLC25A5 |  |  | 2796 | 0.290 | 0.3237 | No |
| 191 | VDAC3 |  |  | 2803 | 0.289 | 0.3239 | No |
| 192 | BUB3 |  |  | 2806 | 0.288 | 0.3249 | No |
| 193 | PRPSAP1 |  |  | 2812 | 0.288 | 0.3253 | No |
| 194 | CBX3 |  |  | 2851 | 0.284 | 0.3191 | No |
| 195 | SAE1 |  |  | 2872 | 0.283 | 0.3165 | No |
| 196 | REEP4 |  |  | 2902 | 0.278 | 0.3121 | No |
| 197 | PPP1CC |  |  | 2903 | 0.278 | 0.3135 | No |
| 198 | BCL2L12 |  |  | 2968 | 0.272 | 0.3021 | No |
| 199 | TNFAIP8L1 |  |  | 3081 | 0.261 | 0.2812 | No |
| 200 | EIF5 |  |  | 3375 | -0.278 | 0.2246 | No |
| 201 | COMMD6 |  |  | 3394 | -0.280 | 0.2224 | No |
| 202 | GOT1 |  |  | 3406 | -0.283 | 0.2216 | No |
| 203 | RBM8A |  |  | 3571 | -0.311 | 0.1906 | No |
| 204 | GLIPR2 |  |  | 3582 | -0.312 | 0.1901 | No |
| 205 | EEF1A1 |  |  | 3617 | -0.318 | 0.1849 | No |
| 206 | GLRX5 |  |  | 3629 | -0.320 | 0.1843 | No |
| 207 | C4orf3 |  |  | 3884 | -0.364 | 0.1358 | No |
| 208 | TUBB6 |  |  | 3904 | -0.368 | 0.1338 | No |
| 209 | EEF2 |  |  | 3913 | -0.369 | 0.1340 | No |
| 210 | CCT5 |  |  | 3958 | -0.379 | 0.1271 | No |
| 211 | LDHA |  |  | 4145 | -0.421 | 0.0923 | No |
| 212 | DDIT4 |  |  | 4182 | -0.432 | 0.0873 | No |
| 213 | ATP6V0E1 |  |  | 4302 | -0.473 | 0.0660 | No |
| 214 | MORF4L2 |  |  | 4354 | -0.488 | 0.0583 | No |
| 215 | IFITM3 |  |  | 4424 | -0.509 | 0.0471 | No |
| 216 | OST4 |  |  | 4463 | -0.524 | 0.0421 | No |
| 217 | FTH1 |  |  | 4515 | -0.547 | 0.0346 | No |
| 218 | IGFBP7 |  |  | 4622 | -0.604 | 0.0165 | No |
| 219 | RPL34 |  |  | 4660 | -0.624 | 0.0122 | No |
| 220 | CDC42EP3 |  |  | 4669 | -0.627 | 0.0137 | No |
| 221 | RPL31 |  |  | 4779 | -0.697 | -0.0046 | No |
| 222 | RAB13 |  |  | 4786 | -0.701 | -0.0024 | No |
| 223 | MT2A |  |  | 4799 | -0.714 | -0.0013 | No |
| 224 | PDLIM7 |  |  | 4867 | -0.773 | -0.0108 | No |
| 225 | C4orf48 |  |  | 4883 | -0.789 | -0.0100 | No |
| 226 | CLIC1 |  |  | 4899 | -0.809 | -0.0091 | No |
| 227 | PLIN3 |  |  | 4921 | -0.833 | -0.0092 | No |
| 228 | C12orf75 |  |  | 4940 | -0.858 | -0.0087 | No |
| 229 | CST3 |  |  | 4951 | -0.868 | -0.0065 | No |
| 230 | CALD1 |  |  | 4960 | -0.877 | -0.0038 | No |
| 231 | HMGA1 |  |  | 4962 | -0.878 | 0.0002 | No |
| 232 | IGFBP5 |  |  | 4978 | -0.897 | 0.0015 | No |
| 233 | ADA |  |  | 5005 | -0.945 | 0.0009 | No |
| 234 | CTGF |  |  | 5070 | -1.073 | -0.0066 | No |
| 235 | FTL |  |  | 5072 | -1.077 | -0.0016 | No |
| 236 | FILIP1L |  |  | 5103 | -1.158 | -0.0019 | No |
| 237 | PRDX1 |  |  | 5104 | -1.163 | 0.0037 | No |
| 238 | GLIPR1 |  |  | 5109 | -1.177 | 0.0085 | No |
| 239 | LMNA |  |  | 5141 | -1.277 | 0.0085 | No |
| 240 | SERPINF1 |  |  | 5158 | -1.351 | 0.0119 | No |
| 241 | SERPINE1 |  |  | 5161 | -1.377 | 0.0181 | No |
| 242 | MIAT |  |  | 5225 | -1.704 | 0.0139 | No |
Table: GSEA details [plain text format]

  

Fig 2: FELDMAN\_2020\_G2-M: Random ES distribution      
 Gene set null distribution of ES for **FELDMAN\_2020\_G2-M**
